# Processing and Comprehension of Locally Ambiguous Participial Relative Clause Sentences in Russian

**DOI:** 10.1007/s10936-024-10041-4

**Published:** 2024-02-21

**Authors:** Liubov Darzhinova, Zoe Pei-sui Luk

**Affiliations:** 1 Department of English Language Education, Faculty of Humanities, The Education University of Hong Kong, 10 Lo Ping Road, Tai Po, New Territories, Hong Kong, China; 2 Department of Linguistics and Modern Language Studies, Faculty of Humanities, The Education University of Hong Kong, Hong Kong, China

**Keywords:** Sentence processing, Syntactic ambiguity, Self-paced reading, Attachment preference, Comprehension

## Abstract

The study tested how the Recency Preference and Predicate Proximity model (Gibson et al. in Cognition 59(1):23–59, 1996, 10.1016/0010-0277(88)90004-2) plays out by examining the attachment preferences of native Russian speakers when processing locally ambiguous participial relative clause sentences with three potential NP attachment sites in Russian. Using a self-paced reading task, reading times and noun phrase selection responses were collected. Results showed significantly shorter reading times at the disambiguating region and higher accuracy rate of selection in the high-attaching condition than in the middle- and low-attaching conditions. No significant differences were found between the middle- and low-attaching conditions. We argue that Predicate Proximity is a much stronger factor than Recency Preference in Russian.

## Introduction

Ambiguities are common in language, and comprehenders are often required to resolve them. The Garden Path theory (Frazier & Fodor, 1978) stipulates that at first, a single syntactical structure is regarded for a sentence, whereas non-structural information, such as semantics, frequency, and discourse contexts, does not impact the processing of a syntactical structure. One principle that is said to be universally operative in this stage of language processing is the Late Closure principle (Frazier, 1979), which states that incoming lexical items tend to be associated with the phrase or clause currently being processed. For example, when processing the sentence in (1), *yesterday* is more likely to be associated with *left* than the matrix verb *said*. Even though both interpretations are possible, the former interpretation requires less processing cost for the memory system than the latter.

**Table:** 

(1)	John said Mary left yesterday

In the case of the processing of relative clauses, this would mean attaching the relative clauses to the closest noun phrase (NP). For example, the Late Closure principle would predict that the relative clause *who punched himself* in (2) modifies *the king* rather than *the son*.

**Table:** 

(2)	The son of the king who punched himself feels embarrassed

Another factor, Predicate Proximity, proposed by Gibson et al. (1996), states that incoming structures are preferred to be placed as close to the head of the predicate phrase as possible. Gibson and colleagues argue that the principles of Late Closure and Predicate Proximity are operative in processing relative clauses.

The objective of this study is to test how these principles play out in the processing of participial relative clause sentences in Russian by native Russian speakers. The paper is organized as follows. First, we review the most classic principles and models advanced in the attachment ambiguity resolution literature to lay out the theoretical and conceptual groundwork for the study. Second, we present an overview of the syntactic ambiguity in Russian, centering on the relative clause construction and the construction employed in this study, i.e., the participial relative clause. Third, we describe the methodology and state our hypotheses. Lastly, we present the findings of the experiment and interpret them in terms of the two-factor model.

### Recency Preference and Predicate Proximity

**Fig. 1:**
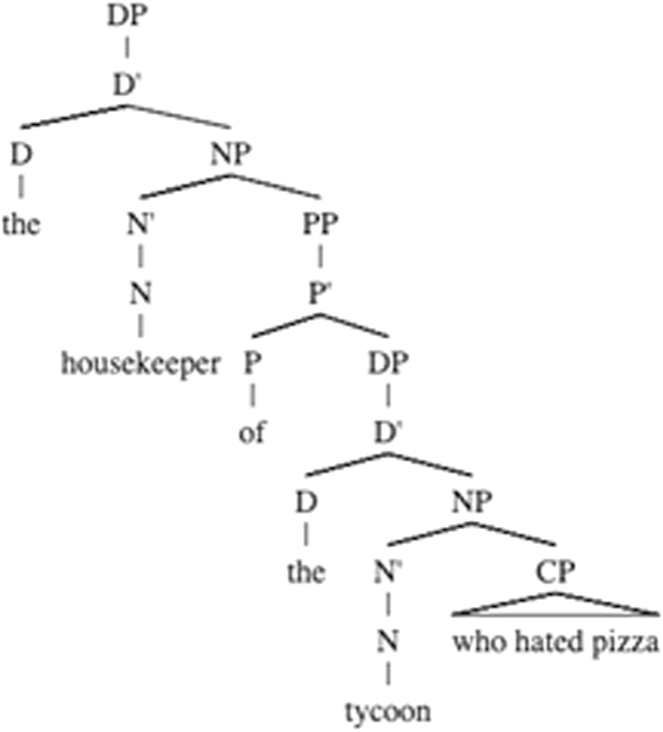
The example of the Recency Preference dominance in resolution of a relative clause attachment ambiguity with a two-site context

As a variant of Late Closure, the Recency Preference (Gibson, 1991) would predict that in the sentence as in (3), the relative clause *who hated pizza* modifies the second noun phrase (NP_2_), *the tycoon* (Fig. 1) instead of the NP_1_, that is higher up in the hierarchy, *the housekeeper*. At the same time, Recency Preference makes more specific predictions than Late Closure, as it lines up the potential attachment sites in the order of processing load (Pearlmutter & Gibson, 2001).

**Table:** 

(3)	The burglar stabbed the housekeeper of the tycoon who hated pizza

Although the property of Recency Preference (or Late Closure) has been demonstrated in the processing of relative clauses in many languages, e.g., English (Fernández, 2003), Arabic (Quinn et al., 2000), Basque (Gutierrez-Ziardegi et al., 2004), Norwegian, Romanian, and Swedish (Ehrlich et al., 1999), it does not seem to apply in some languages. Cuetos and Mitchell (1988) showed that native Spanish speakers prefer attaching a relative clause to the first noun instead of the second when there are two potential sites, i.e., ‘the daughter’ in (4). The authors argued that the results suggested that Late Closure is not a universally operative principle.

**Table:** 

(4)	*El*	*periodista*	*entrevistó*	*a*	*la*	*hija*	*del*	*coronel*	*que*	*tuvo*	*el*	*accidente*
	‘The journalist interviewed the daughter of the colonel who had the accident.’											

Gibson et al. (1996) argue that the effect of Recency Preference might actually exist in Spanish, but it could be modulated by another factor, which they termed Predicate Proximity (Gibson et al., 1996, p. 23). Predicate Proximity predicts that a relative clause should be attached “as close as possible to the head of the predicate phrase” (Gibson et al., 1996, p. 41). This property is motivated by the fact that the argument(s) of a verb in a sentence is/are of greater importance than non-argument NPs, and therefore, it will be more available for attachment than other potential sites.

To verify this two-factor model, Gibson and colleagues used sentences with relative clauses involving three possible attachment sites, as in (5a). Using the self-paced reading task, they analyzed these sentences’ grammaticality judgments and reading times. The attachment predisposition to high, middle, and low attachments was controlled by one of the preceding NPs agreeing with the verb in the relative clause in number so that, for instance, the middle-attaching version looked like (5b), in which only the picture agrees in number with the auxiliary verb *was*. They hypothesized that if there is only one factor at play, the attachment preference should show only an increasing (i.e., the lamp < the picture < the villa) or decreasing trend (i.e., the lamp > the picture > the villa). If, however, both Recency Preference and Predicate Proximity are involved in the processing, then the results should show a higher preference for the NP_1_ and the NP_3_ than the NP_2_ because neither of the two factors is relevant to the NP_2_.

**Table:** 

(5)	a	The	lamp	near	the	picture	of	the	villa	which	was	damaged	by	fire
	b	The	lamps	near	the	picture	of	the	villas	which	was	damaged	by	fire

In the first experiment, they tested the processing of these sentences in Spanish by native Spanish speakers. The participants were asked to press the space button to read sentences in a word-by-word fashion. They could press the button specified ‘NO’ at any point whenever they deemed the sentence unacceptable in Spanish. Results showed that the low-attaching version was judged as ungrammatical less often than the middle- or high-attaching versions, whereas the high-attaching version was judged as ungrammatical less often than the middle-attaching version. As for the reading times, the disambiguating region, that is, the auxiliary verb *fue* ‘be’, in the low-attaching version was processed faster than in any of the other versions, while it was processed faster in the high-attaching version than in the middle-attaching version. They argue that these results suggest that the processing is influenced by two factors, one causing robust preference to attach a relative clause low and the other facilitating the processing of the high-attaching version.

In the second experiment, they tested native English speakers with the English versions of the sentences used in the first experiment. Results showed that the low-attaching version was judged as ungrammatical less often than the middle-attaching or high-attaching versions, but the difference in ungrammaticality between the latter two conditions did not reach significance. The disambiguating region in the low-attaching version was read faster than in either of the other two versions, while it was read faster in the high-attaching version than in the middle-attaching version. Even though ungrammaticality judgment data did not show any clear pattern, the reading time data suggest that the middle-attaching version was processed more slowly than both high- and low-attaching versions, indicating more difficulty in attaching a relative clause middle than to the low and high-attaching sites. The authors argue that the results of the two experiments suggest that both the Recency Preference factor and the Predicate Proximity factor are operative in processing relative clauses in English and Spanish.

The two-factor model has been shown to predict attachment preferences in other head-initial languages, such as German (Walter & Hemforth, 1998), Dutch (Wijnen, 1998), and Japanese (Miyamoto et al., 1999), which is a head-final language. In this study, we attempted to extend the investigation to participial relative clauses in Russian. The rationale for choosing participial relative clauses as the study material is that the morphological complexity of the participle may cause differences in processing strategies. In the following section, we describe the characteristics of relative and participial relative clauses in Russian.

### Relative and Participial Relative Clauses in Russian

In Russian, relative clauses and participial relative clauses are often viewed as synonymous constructions because both can express the same meaning and, under certain conditions, can replace each other. They are sometimes used interchangeably to avoid repetition of relative pronouns or participles in the chain of sentences.

The participle in Russian is considered either a non-finite form of the verb, a special form of the adjective, or an independent part of speech. Participles combine the morphological features of an adjective and a verb. The features of the verb in participles are such categories as aspect, tense, transitivity, and voice. Participles convey the meaning of process (processuality), e.g., the participle *krashennyy* ‘painted’ as in the phrase (6) stresses the processuality and not the quality of the noun it modifies, cf. *nekrashenyy zabor* ‘an unpainted fence.’ At the same time, participles are comparable to adjectives in morphological properties—they agree in gender, number, and case with the nouns they modify. Compared to the finite verb form that is used in a full relative clause, the participle form is more complex as it has an additional morphological complexity that transforms the verb into a participle and marks aspect and voice.

**Table:** 

(6)	*zabor,*		*krash-enn-yy*
	fence.MASC.NOM.SG		painted-PTCP.IPFV.PASS-MASC.NOM.SG
	*nami*	*proshlym*	*letom*
	by.us	last	summer
	‘fence painted by us last summer’		

It must be noted that participial relative clauses cannot be used to express future events. The primary function of participles is to indicate an actual event that has already occurred or is currently taking place (Devyatova, 2013).

A participial clause, as in (7a), and a relative clause, as in (8), sometimes permit double modification (Rozental et al., 1999), causing global syntactic ambiguity. For example, it is unclear whether *predsedatel’* ‘the chairman’ or *komitet* ‘the committee’ deals with the issues.^1^ The sentence (7a) would be free of global syntactic ambiguity if paraphrased towards high attachment through morphological agreement in the instrumental case between the participle and the NP_1_, such as in (7b), or towards low attachment through morphological agreement in the genitive case between the participle and the NP_2_, such as in (7c).

**Table:** 

(7)	a	*Zayavleni-ye*		*predsedatel-ya*	
		Statement-N.NOM.SG		chairman-MASC.GEN.SG	
		*komitet-a*,			
		committee-MASC.GEN.SG,			
		*zanima-yushch-ego-sya*			*etimi*
		deal-PTCP.IPFV.PRS.ACT.INTR.MASC.SG-GEN-REFL			these
		*voprosami,*	*vyzvalo*	*rezonans*	
		questions,	cause	resonance	
		‘The statement of the chairman of the committee dealing with these issues caused a resonance.’			
	b	*Zayavleni-ye*		*sdelann-oye*	
		Statement-N.NOM.SG		make-PTCP.PFV.PST.PASS.INTR.N.NOM.SG-NREFL	
		*predsedatel-em*		*komitet-a,*	
		chairman-MASC.INS.SG		committee-MASC.GEN.SG,	
		*zanima-yushch-im-sya*			*etimi*
		deal-PTCP.IPFV.PRS.ACT.INTR.MASC.SG-INS-REFL			these
		*voprosami,*	*vyzvalo*	*rezonans*	
		questions,	cause	resonance	
		‘The statement made by the chairman of the committee dealing with these issues caused a resonance. The chairman dealt with these issues.’			
	c	*Zayavleni-ye*		*sdelann-oye*	
		Statement-N.NOM.SG		make-PTCP.PFV.PST.PASS.INTR.N.NOM.SG-NREFL	
		*predsedatel-em*		*komitet-a,*	
		chairman-MASC.INS.SG		committee-MASC.GEN.SG,	
		*zanima-yushch-ego-sya*			*etimi*
		deal-PTCP.IPFV.PRS.ACT.INTR.MASC.SG-GEN-REFL		these	
		*voprosami,*	*vyzvalo*	*rezonans*	
		questions,	cause	resonance	
		‘The statement made by the chairman of the committee dealing with these issues caused a resonance. The committee dealt with these issues.’			

**Table:** 

(8)	*Zayavleni-ye*	*predsedatel-ya*	*komitet-a,*	
	Statement-N.NOM.SG	chairman-MASC.GEN.SG	committee-MASC.GEN.SG,	
	*kotor-yy*	*zanima-yet-sya*		
	which(who)-MASC.GEN.SG	deal-INTR.IND-IPFV.MASC.GEN.SG-REFL		
	*etimi*	*voprosami,*	*vyzvalo*	*rezonans*
	these	questions,	cause	resonance
	‘The statement of the chairman of the committee, which deals with these issues caused a resonance.’			

Replacing a relative clause with a participial one may change the stylistic coloring and semantics. For example, when it is necessary to emphasize the meaning of the subject’s action, participial relative clauses are regarded as inferior to the relative clauses in their expressiveness (Petukhova & Simulina, 2015). Participial relative clauses are used more often in academic writing than relative clauses. Another difference between participial relative clauses and relative clauses is that the former can be introduced before or after the constituent they modify, whereas relative clauses can only be placed after that constituent (Koprov, 2019; Zhurbina & Melkumyants, 2010).

### Syntactic Ambiguity in Russian

The issue of syntactic ambiguity in Russian has been investigated on the material of relative clause attachment ambiguity in locally ambiguous sentences with the two-site (Sekerina, 2003) and three-site (Dragoy, 2006) contexts. There has also been a surge of interest in attachment ambiguity instantiated in locally and globally ambiguous participial relative clause sentences with the two-site context (Chernova & Chernigovskaya, 2015).

Sekerina (2003) conducted a study to test whether the low attachment of the relative clause to the complex NP applies to the Russian language. In Experiment 1, participants read globally ambiguous relative clause sentences as in (9) and sentences that feature a lexical preposition *k* ‘to’ in (10) in the off-line rating acceptability questionnaire and were given two interpretations, a high-attaching interpretation (e.g., ‘The illustrations were performed professionally’) and a low-attaching interpretation (e.g., ‘The stories were performed professionally’). They were instructed to indicate the plausibility of each interpretation on a scale from 0 to 3, with 0 being *very implausible* and 3 being *very plausible*. Results showed that, regardless of whether there was a lexical preposition, participants rated the high-attaching interpretation significantly higher than the low-attaching interpretation. Experiment 2, which required participants to read sentences under time constraints, revealed similar results.

**Table:** 

(9)	*Nikolay*	*khorosho*	*zna-l*	*syn-a*
	Nikolay	well	know-PST.MASC.SG	son-MASC.ACC.SG
	*polkovnik-a*		*kotor-yy*	*po-gib*
	colonel-MASC.GEN.SG		who-MASC.NOM.SG	PFV-kill-MASC.SG
	*v*	*avtomobilʹn-oy*	*katastrophe*	
	in	car	accident	
	‘Nikolay knew well the son of the colonel who was killed in a car accident.’			

**Table:** 

(10)	*Illyustratsi-i*	*k*	*rasskaz-am*	
	Illustrations-NOM.PL	to	stories-DAT.PL	
	*kotor-yye*	*by-l-i*	*pri-sl-an-y*	*na*
	which-NOM.PL	are-PST-PL	PVF-send-PST-PL	to
	*konkurs,*	*ispoln-en-y*		*masterski*
	contest	perform-PTCP.PASS-PL		professionally
	‘The illustrations to the stories which were sent to the contest are performed professionally.’			

Dragoy (2006) tested the effect of working memory capacity on attachment preference. The recruited participants were native speakers who were university students at the time of the experiment. The material involved Russian relative clause sentences with three attachment sites, as in (11).

**Table:** 

(11)	*Slozhno*	*ponyat’*	*logik-u*
	Difficult	understand	logic-FEM.GEN.SG
	*organizatsi-i*	*rech-i,*	*kotor-a-ya*
	organization-FEM.GEN.SG	speech-FEM.GEN.SG,	which-FEM.NOM.SG
	*narush-en-a*		
	damage-PTCP.PASS-FEM.SG		
	‘It is difficult to understand the logic of the organization of speech, which is damaged.’		

The participants were instructed to read aloud the experimental sentences appearing on the computer screen. They were then presented with three interpretations, e.g., ‘logic’, ‘organization’, ‘speech’ in (11). They were asked to choose one option, say it aloud, and simultaneously press the key corresponding to that option. The results showed that the high attachment interpretation was the most preferred by the low working memory capacity group, followed by low and middle attachment. For the group with high working memory capacity, low attachment interpretation prevailed over high attachment interpretation. This result contradicts the belief that Russian prefers high attachment. The group with medium working memory capacity showed no significant differences between the high and low attachment interpretations, with significantly less preference for the middle attachment interpretation. The findings suggest the simultaneous activation of the Predicate Proximity and Recency Preference factors, which, however, have different strengths in groups with different levels of working memory capacity.

Chernova and Chernigovskaya (2015) employed the self-paced reading (Experiment 1) and eye-tracking (Experiment 2) paradigms. Their material contained Russian globally ambiguous participial relative clause sentences with two possible NP attachment sites, such as in (12), and locally ambiguous sentences of the same type, disambiguated by case, as in (13) and (14). Experiment 1 showed that low-attaching sentences were misread as high-attaching more often than high-attaching sentences as low-attaching, suggesting that high attachment effects are robust. The sentences in the low-attaching version were also comprehended less accurately than those in the high-attaching version. Globally ambiguous sentences were read as high-attaching more frequently than low-attaching. However, sentences with a low attachment interpretation were processed faster than those with a high attachment interpretation. This result was unexpected, given that high attachment is the preferred option. The authors interpreted these reading time results as influenced by the Late Closure principle (Frazier & Fodor, 1978). The author argued that high attachment demands more working memory resources and takes longer to process than low attachments because the agreement between a noun and a participle is not local (cf. Swets et al., 2007).

**Table:** 

(12)	*Svidetel’*	*u-pomyanu-l*	
	Witness-MASC.NOM.SG	PFV-mention-PST.MASC.SG	
	*naparnik-a*	*voditel-ya*	*vchera*
	workmate-MASC.GEN.SG	driver-MASC.GEN.SG	yesterday
	*vide-vsh-ego*	*eto*	*ogrableniye*
	see-PTCP.PFV.ACT-MASC.GEN.SG	this	robbery
	‘The witness mentioned the workmate of the driver yesterday having seen this robbery.’		

**Table:** 

(13)	*Svidetel’*	*u-pomyanu-l*	*o*
	Witness-MASC.NOM.SG	PFV-mention-PST.MASC.SG	about
	*naparnik-e*	*voditel-ya*	*vchera*
	workmate-MASC.PREP.SG	driver-MASC.GEN.SG	yesterday
	*vide-vsh-ego*	*eto*	*ogrableniye*
	see-PTCP.PFV.ACT-MASC.GEN.SG	this	robbery
	‘The witness mentioned about the workmate of the driver yesterday having seen this robbery. The driver saw this robbery.’		

**Table:** 

(14)	*Svidetel’*	*u-pomyanu-l*	*o*
	Witness-MASC.NOM.SG	PFV-mention-PST.MASC.SG	about
	*naparnik-e*	*voditel-ya*	*vchera*
	workmate-MASC.PREP.SG	driver-MASC.GEN.SG	yesterday
	*vide-vsh-em*	*eto*	*ogrableniye*
	see-PTCP.PFV.ACT-MASC.PREP.SG	this	robbery
	‘The witness mentioned about the workmate of the driver yesterday having seen this robbery. The workmate saw this robbery.’		

Experiment 2, which was an eye-tracking experiment, found that regressions to or from the participle region were made more often for low-attaching and globally ambiguous sentences than for high-attaching sentences. A rereading of the NP_1_ region occurred twice more often than of the NP_2_ region. These results matched the comprehension results, showing that global ambiguity is resolved more towards high than low attachments. The off-line comprehension accuracy rates obtained in Experiment 1 of the study provide some evidence for high attachment preference in native Russian speakers, even though attaching high requires more time.

Chernova and Prokopenya (2016) administered a story-continuation task (15) to native speakers of Russian to test whether contextual expectations influence the resolution of syntactic ambiguity. The participants were asked to fill in the blanks with whatever came to mind first. It was found that most participants responded in a way that did not resolve the ambiguity and maintained it by producing third-person pronouns, e.g., *ona* ‘she’. Other participants put the NP_1_ or their synonyms in the blank, whereas the NP_2_ or its periphrases were the least common responses. The authors concluded that even though most participants did not resolve ambiguity by filling in the blank with the pronoun ‘she’, variations of the NP_1_ filling-in-the-blank were the second most predominant. This combination of findings was taken as an indication of high attachment preference in Russian. The authors also suggested that readers avoid resolving ambiguity as they are driven by the so-called egocentric approach (Kibrik, 2011). This approach represents the case when one maintains the tacit assumption that others’ mental processes align with theirs, so there is no need to specify who the referent is, e.g., the servant-FEM or the countess-FEM.

**Table:** 

(15)	*Ya*		*vstre-ti-l-(a)*	*sluzh-ank-u*	
	I		meet-PFV-PST-MASC(FEM).SG	servant-ACC.FEM-SG	
	*graf-in-i*			*na*	*ulitse*
	countess-GEN.FEM-SG	on	street		
	‘I met the servant of the countess on the street.’				
	*Mnogo*	*let*	*________*	*zhi-l-a*	*ryadom*
	Many	year	________	live-PFV.PST-SG.FEM	nearby
	‘For many years ____ had been living nearby.’				

To sum up, there is a predominance of research examining how native Russian speakers connect a relative clause to one of the two/three NP attachment sites and a participial relative clause to one of the two sites. These results, in general, suggest a preference for high attachment in Russian, except for the native Russian-speaking participants who had high working memory capacity in Dragoy (2006). However, the general parsing strategy applicable to the participial relative clause construction having three possible attachment sites has not been studied. Thus far, no study has employed an on-line task to investigate the processing of participial relative clauses with three potential attachment sites. A morphosyntactically different structure could generate different results. For example, Pearlmutter and Gibson (2001) found a monotonic recency-based attachment preference ordering for attaching time adverbials (e.g., *yesterday*) and infinitival purpose clauses to verb phrases, contradicting the non-monotonic results found in relative clauses in English. Therefore, we extended the investigation to participial relative clauses in Russian, given their divergence in morphosyntactic features from full relative clauses. We predicted that the additional morphological complexity of the participle form could cause differences in processing loads and, thus, attachment preferences. The study fills the gap by testing how native Russian speakers process and comprehend sentences featuring this construction.

## Methods

### Participants

Thirty-five monolingual speakers of Russian were recruited for this study. Nine participants showed less than 70% comprehension accuracy of filler sentences (to be further explained in the Procedures section), and their data were not included in the analyses. As a result, the data of 26 participants were analyzed. The biographic information of the participants was confirmed verbally with the investigator, who is the first author of this paper. The recruited participants were permanent residents of Russia who had no or little knowledge of other languages, including regional languages (e.g., Oirat-Kalmyk, Buryat, and Tatar). None of the participants had any training in linguistics. All participants had normal or corrected-to-normal vision and reported no reading difficulties.

### Material and Design

The experimental design was adapted from Gibson et al. (1996). Participial relative clause sentences with three potential NP attachment sites were used as the experimental items. All the experimental items followed the template: Subject verb NP_1_ NP_2_ NP_3_ participial relative clause. The experimental items were designed such that only one of the three NPs agreed in number with the participle in the participial relative clause. In other words, only one reading is grammatical. For example, the participial relative clause in (16) is associated with NP_1_ because the participle *zatyan-uvsh-ego-sya* ‘delay-PTCP.PFV.PST.ACT.INTR.N.PASS.**SG**-GEN-REFL’ agrees in number with *ispoln-eni-ya* ‘execution-GEN-N-**SG**.’ The sentences in (17) and (18) are associated with NP_2_ and NP_3_, respectively, for the same reason. All the NPs agreed with the participle in the participial relative clause in the neuter gender, and all participles were singular and in passive voice (i.e., a form equivalent to the past participle in English).

**Table:** 

(16)	*Istts-y*		*potrebov-a-l-i*	*ispoln-eni-ya*
	Plaintiff-NOM.MASC.PL		demand-PFV-PST-PL	execution-N.GEN-SG
	*resh-eniy*	*del,*		
	judgment-GEN.N.PL	case-GEN.N.PL		
	*zatyan-uvsh-ego-sya*			
	delay-PTCP.PFV.PST.ACT.INTR.N.PASS.SG-GEN-REFL			
	*iz-za pandem-i-i*			
	due.to pandemic			
	‘The plaintiffs demanded the execution of the judgments of the cases (the judgments’ execution of the cases) delayed due to the pandemic. The execution was delayed due to the pandemic.’			

**Table:** 

(17)	*Istts-y*		*potrebov-a-l-i*	*ispoln-eni-y*
	Plaintiff-NOM.MASC.PL		demand-PFV-PST-PL	execution-N.GEN-PL
	*resh-eni-ya*	*del,*		
	judgment-GEN.N-SG	case-GEN.N.PL		
	*zatyan-uvsh-ego-sya*			
	delay-PTCP.PFV.PST.ACT.INTR.N.PASS.SG-GEN-REFL			
	*iz-za pandem-i-i*			
	due.to pandemic			
	‘The plaintiffs demanded the executions of the judgment of the cases delayed due to the pandemic. The judgment was delayed due to the pandemic.’			

**Table:** 

(18)	*Istts-y*		*potrebov-a-l-I*		*ispoln-eni-y*
	Plaintiff-NOM.MASC.PL		demand-PFV-PST-PL		execution-N.GEN.PL
	*resh-eniy*	*del-a,*			
	judgment-GEN.N.PL	case-GEN.N-SG			
	*zatyan-uvsh-ego-sya*				
	delay-PTCP.PFV.PST.ACT.INTR.N.PASS.SG-GEN-REFL				
	*iz-za pandem-i-i*				
	due.to pandemic				
	‘The plaintiffs demanded the executions of the judgments of the case delayed due to the pandemic. The case was delayed due to the pandemic.’				

The experimental items were also designed such that the NPs are equally pragmatically compatible. For instance, the three NPs *ispolnenie* ‘execution’, *resheniye* ‘judgment’, or *delo* ‘case’ were semantically compatible with ‘delayed due to the pandemic.’ Animacy of antecedents was controlled for, with all the NPs being inanimate. The discourses in experimental sentences were inspired by textual materials in Russian that are freely available on the Internet, such as official documents, news articles, and excerpts from books.

To ensure the naturalness and plausibility of the material, a norming study was carried out before the actual experiment. Ten native speakers of Russian were invited via phone conversation to evaluate the sentences. The norming study was coordinated via e-mail or mobile messengers. Each rater was provided with a list of sentences in a digital file. Since three versions with different attachment options were created for each context, all three versions were sent to the raters for evaluation. They were asked to rate their plausibility and naturalness by putting a plus (+) or minus (−) symbol next to each sentence to indicate whether that sentence sounds plausible and natural or implausible and unnatural, respectively. Sentences that were marked as unnatural and implausible by most raters were replaced or modified and then again underwent another round of the norming procedure until all of them were deemed natural.

The experiment featured 18 different sets (contexts), each consisting of 3 sentences, resulting in a total of 54 sentences across all conditions: the NP_1_ condition (16), the NP_2_ condition (17), and the NP_3_ condition (18). In addition, 60 filler sentences were included as distractors to ensure that participants did not become accustomed to the experimental sentences (Rah & Adone, 2010). Another function of these filler sentences was to test the participants’ sensitivity to morphological marking. The set of fillers included 30 grammatical globally ambiguous relative clause sentences with two potential NP attachment sites (19) and 30 ungrammatical relative clause sentences with two NP attachment sites (20), which were ungrammatical because neither of the NPs agreed in gender with the relative pronoun and the verb. The fillers were similar in length and semantic complexity to the test sentences.

**Table:** 

(19)	*Zhyuri*	*diskvalifitsirovalo*	*sopernik-a*	
	Jury	disqualified	rival-GEN.MASC.SG	
	*atlet-a,*		*kotor-yy*	*gotov-il-sya*
	athlete-GEN.MASC.SG		who-GEN.MASC.SG	prepare-MASC.SG.PST-REFL
	*k*	*sorevnovaniyam*		
	for	competition		
	‘The jury disqualified the rival of the athlete who was preparing for the competition.’			

**Table:** 

(20)	*Voditel’*	*lishilsya*	*dvigatel-ya*	*avtomobil-ya,*
	Driver	lost	engine-GEN.MASC.SG	car-GEN.MASC.SG,
	*kotor-oye*	*by-l-o*	*neispravno*	
	which-N.SG	is-PST-N.SG	defective	
	‘The driver lost the engine of the car, which was defective.’			

Four practice sentences were given to the participants at the beginning of the experiment to familiarize them with the task. The test sentences were counterbalanced across three lists so that each list contained sentences in all three conditions, with each context appearing only once in each list. The filler sentences were the same for all the lists. As a result, each participant saw a total of 82 sentences, including four practice sentences, 18 test sentences, and 60 filler sentences.

### Procedures

All participants gave informed consent prior to the procedure. Before starting the experiment, they were asked to complete the web-based version of the Edinburgh Handedness Inventory (Oldfield, 1971) to ascertain their handedness, which would determine which set of buttons they would use during the self-paced reading experiment. All the participants were right-handed.

The self-paced reading task in a non-cumulative fashion (moving-window) was developed in the customized PsychoPy script (Peirce et al., 2019). Each experimental sentence was divided into seven regions for further analyses, as in (21), whereas filler sentences were split into six regions, as in (22) and (23). As for the experimental sentences, the first region (R1) consisted of a subject and a verb. The subject was always animate and in plural form. R2, R3, and R4 consisted of NP_1_, NP_2_, and NP_3_, respectively, all of which were inflected for gender and number. R5 consisted of the participle that was inflected for number, where the participants could decide which NP the participial relative clause modified. Therefore, R5 served as the disambiguating region. R6 and R7 consisted of the remainder of the sentence.

**Table:** 

(21)							

**Table:** 

(22)						

**Table:** 

(23)						

The task also incorporated comprehension questions to elicit off-line data about ungrammaticality judgment. The purpose of having the comprehension questions was to check whether the participants paid attention to gender and number morphology. Each participant’s comprehension accuracy of the filler sentences was checked to ensure that the participants read the sentences attentively during the procedure. The data of the participants, whose accuracy rate did not reach 70%, were excluded from the analyses.

The experiment was conducted using the first author’s laptop with a 15.6-inch display. Each participant saw the welcome screen with instructions, followed by four practice sentences. The participants were then shown a set of experimental (*n* = 18) and filler (*n* = 60) sentences in a randomized fashion. At the beginning of each trial, a white asterisk was visible in the center of the black screen. When participants pressed the spacebar, the first region of the item was displayed. When the space bar was pressed a second time, the first region disappeared, and the second region appeared in place. Each subsequent space bar press disclosed the following region and removed the prior region. The program recorded the time between all the space bar presses.

Pressing the space bar on the last region of each item triggered the item to be superseded by a new screen with a comprehension question (e.g., ‘What was delayed due to the pandemic?’). The same screen displayed three response options beneath the question. Participants had to select the antecedent by pressing one of the buttons marked ‘NP_1_’, e.g., ‘execution’, or ‘NP_2_’, e.g., ‘judgment’, or ‘NP_3_’, e.g., ‘case.’ There was no time restriction for responding to the question. If they deemed that the read sentence was ungrammatical or unacceptable in Russian, they had to press the button marked ‘ungrammatical/unacceptable’. Right-handed participants used the buttons ‘3’, ‘2’, ‘1’, and ‘SPACE’ for NP_1_, NP_2_, NP_3_, and ‘ungrammatical’, respectively. ‘0’, ‘−’, ‘ = ’ were meant for left-handed participants, but there were no such participants. The experiment took about 22 min on average.

### Data analyses

Following Gibson et al. (1996), our study gathered both on-line and off-line measures, namely the reading times and the ungrammaticality judgments. Effects were expected to be found where the attachment of the participle is disambiguated (i.e., R5). In this study, we also analyzed the accuracy rate of NP selection.

The reading times were recorded region-by-region during the self-paced reading of the participants for subsequent analyses. The data points of the experimental sentences judged as ungrammatical or unacceptable in Russian were removed because judging a sentence as ungrammatical might mean that the participant did not notice a number agreement between the participle and one of the NPs in pertinent sentences at all. However, data points from trials for which the participants chose an incorrect NP were included. Previous studies on garden-path sentences (e.g., Christianson et al., 2001; Huang & Ferreira, 2021) showed that participants could have interpreted the sentence correctly during on-line processing but gave a wrong interpretation when asked to provide an interpretation off-line. Therefore, we believe that, even though participants gave an incorrect NP during the off-line task, they could have implicitly associated the participial relative clause with the correct NP during on-line processing. Therefore, we chose to include those data points. As a result, 2408 of 3276 observations (i.e., 344 of 468 data points per reading region) remained, which is 74.7% of the collected data.

### Hypotheses

Based on the findings of Gibson et al. (1996), we hypothesized the following:

#### Hypothesis 1

The reading times of the disambiguating region are shorter for the sentences in the NP_1_ condition than in the NP_3_ condition and the NP_2_ condition.

#### Hypothesis 2

The reading times of the disambiguating region are shorter for the sentences in the NP_3_ condition than in the NP_2_ condition.

#### Hypothesis 3

The sentences in the NP_1_ condition are judged as ungrammatical less often than those in the NP_3_ condition and the NP_2_ condition.

#### Hypothesis 4

The sentences in the NP_3_ condition are judged as ungrammatical less often than those in the NP_2_ condition.

#### Hypothesis 5

The sentences in the NP_1_ condition are comprehended more accurately than those in the NP_2_ condition and NP_3_ condition.

#### Hypothesis 6

The sentences in the NP_3_ condition are comprehended more accurately than those in the NP_2_ condition.

The above hypotheses were put forth because we assumed that both factors of Predicate Proximity and Recency Preference would be at play. The force of Predicate Proximity yields high attachment preference. At the same time, the Recency Preference factor favors low attachment. Based on Sekerina (2003) and Chernova and Chernigovskaya (2015), Predicate Proximity is assumed to be stronger than Recency Preference in native Russian speakers. Since there is no force behind the preference to attach middle, participants should produce longer reading times, a higher rate of ungrammaticality, and lower selection accuracy in the NP_2_ condition than in the other two conditions.

## Results

### Reading Times

Reading times that were 2.5 SD below or above the mean from the group mean per each region and per each experimental condition were considered outliers. The cut-off of 2.5 SD is often selected for psycholinguistic experiments with healthy adults to ensure that uncommonly slow or fast reading time resulting from distractions or accidental button pressing does not distort the data (Reifegerste & Felser, 2017). It was also used in previous studies involving sentences with ambiguity (e.g., Carreiras et al., 2010; Narumi & Yokokawa, 2013; Teubner-Rhodes et al., 2016).

**Fig. 2:**
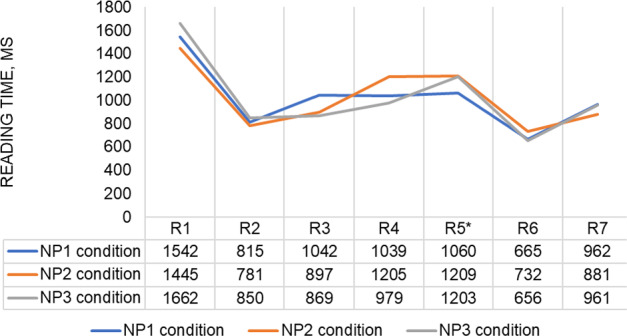
The region-by-region reading times for experimental sentences per condition

These outliers were identified through using the z-score approach, in which standardized values of region-by-region reading times are computed as variables. The actual values were removed at both negative (− 2.5 SD) and positive (2.5 SD) ends. This procedure eliminated 8 data points in the disambiguating region, or 0.33% of all the reading time data points that remained after the prior removal, 5 data points (0.21%) in the Subject + Verb region (i.e., R1) and in the NP_2_ region each, 2 data points (0.08%) in the NP_1_ region, 9 data points (0.37%) in the NP_3_ region, 6 data points (0.25%) in the Remainder region, and 16 data points (0.66%) in the Final Word region. After the second round of data cleaning, 2357 observations were kept for analyses. The SD of the reading times in the disambiguating region changed to 0.526 from 0.813. The mean reading times in the different regions of the experimental sentences in the three conditions are shown in Table 1 and Fig. 2, where the disambiguating region is indicated with an asterisk.

**Table 1:** The mean reading times (SDs) in different regions of the experimental sentences in the three conditions

	NP_1_	NP_2_	NP_3_
R1	1542 (778)	1445 (561)	1662 (791)
R2	815 (415)	781 (419)	850 (416)
R3	1042 (598)	897 (505)	869 (421)
R4	1039 (588)	1205 (723)	979 (491)
R5*	1060 (493)	1209 (657)	1203 (586)
R6	665 (274)	732 (312)	656 (248)
R7	962 (585)	881 (523)	961 (551)

*Critical region

The reading times at the critical, i.e., disambiguating region, were sent to generalized linear mixed effects modeling (GLMM) with the gamma distribution in IBM SPSS Statistics 27, with the attachment predisposition (three levels: NP_1_, NP_2_, and NP_3_) as a fixed effect. Item, Participant, and their interaction term (Item * Participant) were the random effects.

The model revealed a marginal effect of attachment predisposition (F(2, 301) = 2.414, *p* = 0.091). Table 2 shows the model for the reading times with attachment predisposition by number agreement as a fixed effect. The least significant difference (LSD) pairwise multiple comparison test (Table 3) revealed a significant difference between the NP_1_ condition and the NP_3_ condition (*p* < 0.05), with the reading times in the NP_1_ condition being significantly shorter than those in the NP_3_ condition. No significant differences were found between the NP_1_ and the NP_2_ conditions (*p* = 0.106), as well as between the NP_2_ and NP_3_ conditions (*p* = 0.718).

**Table 2:** Fixed coefficients of the GLMM fitted to the reading time data at the disambiguating region

Predictor	β	SE	t	*p*	%CI
Intercept	0.012	0.0698	0.168	0.867	[− 0.130, 0.153]
AttachmentPredisp1	0				
AttachmentPredisp2	0.088	0.0537	1.636	0.103	[− 0.018, 0.194]
AttachmentPredisp3	0.107	0.0516	2.076	0.039	[0.006, 0.209]

**Table 3:** Pairwise contrasts of the GLMM fitted to the reading time data at the disambiguating region

Contrast	Estimate	SE	t	df	*p*	% CI
NP_3_–NP_1_	0.114	0.056	2.055	310	0.041	[0.005, 0.224]
NP_2_–NP_1_	0.093	0.057	1.620	307	0.106	[− 0.020, 0.206]
NP_3_–NP_2_	0.022	0.060	0.361	302	0.718	[− 0.096, 0.139]

### Ungrammaticality Judgments

‘Ungrammatical’ responses were coded as 1, whereas any other responses (i.e., responses indicating a choice among the three NPs) were coded as 0. The means and SDs of ungrammaticality judgments computed from the collected data in each experimental condition for the experimental group are displayed in Table 4.

**Table 4:** Means and SDs of ungrammaticality judgments for each condition of the experiment

	M	SD
NP_1_ condition	0.237	0.427
NP_2_ condition	0.320	0.468
NP_3_ condition	0.237	0.427

The ungrammaticality judgment data were also sent to the GLMM using the binary logistic distribution with the same fixed and random effects. Results did not show a significant effect of attachment predisposition (F(2, 465) = 2.041, *p* = 0.131). Table 5 shows the model for the ungrammaticality judgments with attachment predisposition by number agreement as a fixed effect. The LSD pairwise multiple comparison test reveals marginally significant differences between the NP_1_ and the NP_2_ conditions as well as the NP_2_ and the NP_3_ conditions, both *p* = 0.086, as outlined in Table 6. No significant differences were found between the NP_1_ and the NP_3_ conditions (*p* = 1.000).

**Table 5:** Fixed coefficients of the GLMM fitted to the ungrammaticality judgment data

Predictor	β	SE	t	*p*	%CI
Intercept	− 1.290	0.2657	− 4.855	0.000	[− 1.822, − 0.759]
AttachmentPredisp1	0				
AttachmentPredisp2	0.463	0.2687	1.724	0.085	[− 0.065, 0.992]
AttachmentPredisp3	− 0.000	0.2801	0.000	1.000	[− 0.550, − 0.550]

**Table 6:** Pairwise contrasts of the GLMM fitted to the ungrammaticality judgment data

Contrast	Estimate	SE	t	*df*	*p*	%CI
NP_2_–NP_3_	0.089	0.052	1.718	465	0.086	[− 0.013, 0.190]
NP_2_–NP_1_	0.089	0.052	1.718	465	0.086	[− 0.013, 0.190]
NP_1_–NP_3_	0	0.047	0	465	1.000	[− 0.093, 0.093]

### NP Selection Data

To examine how accurate the participants were in interpreting the sentences when they thought that the sentence was acceptable, we excluded the ‘ungrammatical responses’ and calculated their accuracy rate. The means and SDs of NP selection accuracy from the collected data in each experimental condition for the experimental group are shown in Table 7.

**Table 7:** Means and SDs of NP selection responses for each condition of the experiment

	M	SD
NP_1_ condition	0.642	0.259
NP_2_ condition	0.373	0.215
NP_3_ condition	0.255	0.219

The GLMM was used to investigate the effect of attachment predisposition by number agreement on NP selection responses. Since response choices were either selected or not selected, they were modeled with the binary logistic distribution, with the same fixed and random effects.

The model revealed a main effect of attachment predisposition (F(2, 465) = 14.091, *p* < 0.001). Table 8 shows the model for the NP selection responses with attachment predisposition by number agreement as a fixed effect. The LSD pairwise multiple comparison test revealed significant differences between the NP_1_ condition and the NP_2_ condition (*p* < 0.001) and the NP_1_ condition and the NP_3_ condition (*p* < 0.001), and no difference between the NP_2_ condition and the NP_3_ condition (*p* = 0.302), as outlined in Table 9.

**Table 8:** Fixed coefficients of the GLMM fitted to the NP selection data

Predictor	β	SE	t	*p*	% CI
Intercept	− 0.133	0.2191	− 0.608	0.547	[− 0.575, 0.308]
AttachmentPredisp1	0				
AttachmentPredisp2	− 0.989	0.2499	− 3.958	0.000	[− 1.480, − 0.498]
AttachmentPredisp3	− 1.274	0.2613	− 4.874	0.000	[− 1.787, − 0.760]

**Table 9:** Pairwise contrasts of the GLMM fitted to the NP selection data

Contrast	Estimate	SE	t	*df*	*p*	% CI
NP_2_–NP_3_	0.049	0.047	1.033	465	0.302	[− 0.044, 0.142]
NP_1_–NP_2_	0.221	0.055	4.057	465	0.000	[0.114, 0.328]
NP_1_–NP_3_	0.270	0.054	5.034	465	0.000	[0.165, 0.375]

One-sample *t* tests revealed that participants performed at above chance level (t(25) = 6.076, *p* < 0.001) in the NP_1_ condition but only at chance level in the NP_2_ condition (t(24) = 0.939, *p* = 0.357) and the NP_3_ condition (t(25) = − 1.831, *p* = 0.079).

**Fig. 3:**
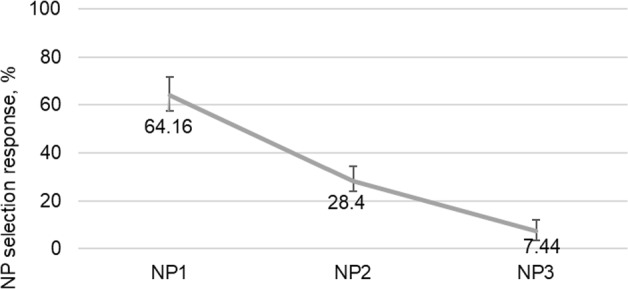
The percentages of NP selection responses for the NP_1_ condition of the experiment

**Fig. 4:**
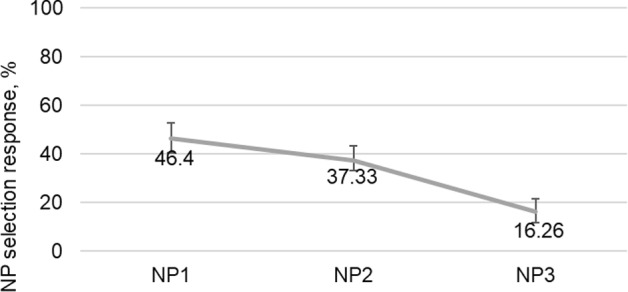
The percentages of NP selection responses for the NP_2_ condition of the experiment

**Fig. 5:**
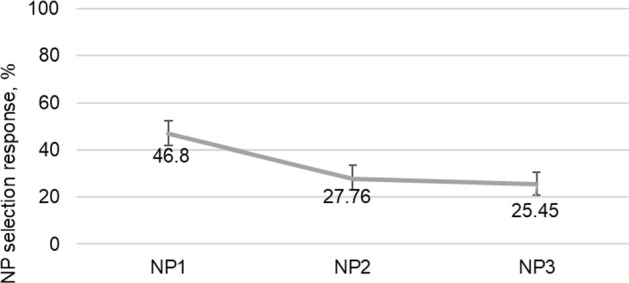
The percentages of NP selection responses for the NP_3_ condition of the experiment

We further examined which of the NPs was chosen in the incorrect NP selection responses per condition. The percentages of the incorrect NP selection responses were normed out of the percentages of the ungrammatical button presses. Figures 3, 4, and 5 present the percentages of NP selection responses per each condition.

## Discussion and Conclusion

The purpose of the study was to test how the two-factor model proposed by Gibson et al. (1996) would play out in the processing of participial relative clauses of Russian. Results show that the reading times at the disambiguating region of native Russian speakers were significantly shorter in sentences in the NP_1_ condition than in the NP_3_ condition but not significantly shorter than in the NP_2_ condition. Hypothesis 1 was only partially confirmed. No significant difference was found in the reading times at the disambiguating region between the NP_2_ and NP_3_ conditions. Hypothesis 2 was therefore not confirmed.

It was also found that the rate of ungrammaticality judgments among native Russian speakers does not significantly differ between the conditions. Therefore, Hypotheses 3 and 4 were rejected. Additionally, it was found that native Russian speakers comprehend the NP_1_ condition significantly more accurately than both the NP_2_ condition and the NP_3_ condition, which confirmed Hypothesis 5. However, sentences in the NP_3_ condition were comprehended not significantly more accurately than those in the NP_2_ condition. Hypothesis 6 was therefore rejected.

These findings suggest that Predicate Proximity is strongly operative during sentence processing in Russian as a first language. We were unable to detect any effect of Recency Preference in this study. There were no significant differences in reading times at the disambiguating region, ungrammaticality judgment, and the rate of NP selection accuracy between the NP_2_ and NP_3_ conditions. Examining the incorrect responses in the NP selection data, we did not find a stronger tendency to choose NP_3_ than NP_2_. We conclude that the Recency Preference is, at most, very weak in Russian, which may not be detectable with this sample size.

The native Russian-speaking participants have an average response accuracy (61.34%) of the high attachment sentences and performed only at chance level in the NP_2_ and NP_3_ conditions. This relatively low response accuracy in the NP_2_ and NP_3_ conditions may be explained by the fact that the task only traces the result of sentence processing, not its processing in real-time. While the participants were at the disambiguating region, they could still be sticking to the high attachment interpretation due to the strong preference for Predicate Proximity, until they noticed that their initial interpretation did not match what the morphological marking suggested. This led to reanalysis and a longer reading time at the region. When the participants were asked to make a judgment, the reanalysis was completed. However, the participants might have had difficulty recalling which noun of the last two nouns morphologically agreed with the participial relative clause, resulting in a low accuracy rate in the NP_2_ and NP_3_ conditions. This finding aligns with Christianson et al. (2001) and Huang and Ferreira (2021), who showed that participants successfully reanalyzed garden-path sentences but were still prone to misinterpretation.

It was also somewhat surprising that the sentences in the NP_2_ and NP_3_ conditions were not judged as ungrammatical more often than those in the NP_1_ condition. This could be because all the experimental items were semantically plausible. When the participants noticed a mismatch in morphological marking with their expectation (i.e., morphological disagreement with NP_1_), they may not jump to the conclusion that the sentence was ungrammatical because the sentence ‘made sense’ to them. Therefore, they attempted to form an interpretation with one of the three nouns, resulting in an ungrammaticality judgment rate similar to that of the NP_1_ condition.

Another note to make is that the accuracy rates were relatively low. In the NP_1_ condition, which was predicted to be the most accessible condition by the participants, the average number of correct responses was only around 3 out of 6. We speculate that this could be due to the complexity of the task, as sentences with participial relative clauses involving three potential attachment sites are rarely seen. The fact that Russian has complex morphological marking might have posed additional processing difficulty. As Christianson et al. (2001) and Huang and Ferreira (2021) have shown, participants who correctly interpret garden-path sentences during on-line processing could still be prone to misinterpretation. The low accuracy rate in our study could be due to similar reasons.

In this study, we were concerned with the question of how strong the Predicate Proximity factor holds relative to the Recency Preference factor; therefore, the inclusion of a third site was necessary to make a contrast between the two factors. We analyzed multiple measures, namely self-paced reading (on-line), ungrammaticality judgments and answers to post-reading comprehension questions (off-line). Taking this approach, we endeavored to get a thorough and holistic picture of how speakers read sentences in real-time, judge their grammaticality, and eventually interpret them. Our findings allowed us to make conclusions about how sentence representations are constructed over time and potentially inform linguistic theory.

To sum up, our experiment renders evidence that the factor Predicate Proximity (Gibson et al., 1996) is strongly operative in the native processing of participial relative clause sentences with three NP attachment sites in Russian. The findings of this study are similar to those of previous research, such as Chernova and Prokopenya (2016) and Sekerina (2003), suggesting a high attachment preference in native Russian speakers. Our study confirmed that the preference for high attachment remains even for participial relative clauses. Dragoy (2006) showed that the attachment preference could be altered by working memory capacity. However, as working memory is not the scope of the current study, future research should examine the effect of working memory capacity.

Our study’s insights into processing and comprehension of locally ambiguous sentences are valuable for the psycholinguistic field that pursues to better understand the mechanisms of parsing decisions and formulate a comprehensive, evidence-based sentence processing model. In future studies, it appears important to analyze whether the high attachment preference is the most frequent interpretation based on Russian corpora. It is particularly interesting to test whether attachment preferences are directly linked with structural frequency and prior linguistic experience.

## Data Availability

The data that support the findings of this study are available from the corresponding author upon reasonable request.

## Footnotes

1 *Zayavleni-ye* ‘statement’ cannot be modified because it is in neuter form and in nominative case, whereas the participle is in masculine form and in genitive case.
